# The Role of Regulatory T Cells in the Biology of Graft Versus Host Disease

**DOI:** 10.3389/fimmu.2013.00163

**Published:** 2013-06-24

**Authors:** Amy J. Beres, William R. Drobyski

**Affiliations:** ^1^Department of Microbiology, Medical College of Wisconsin, Milwaukee, WI, USA; ^2^Department of Medicine, Medical College of Wisconsin, Milwaukee, WI, USA

**Keywords:** graft versus host disease, regulatory T cells, allogeneic stem cell transplantation, induced regulatory T cells, mouse models

## Abstract

Graft versus host disease (GVHD) is the major complication of allogeneic hematopoietic stem cell transplantation. GVHD is characterized by an imbalance between the effector and regulatory arms of the immune system which results in the over production of inflammatory cytokines. Moreover, there is a persistent reduction in the number of regulatory T (Treg) cells which limits the ability of the immune system to re-calibrate this proinflammatory environment. Treg cells are comprised of both natural and induced populations which have unique ontological and developmental characteristics that impact how they function within the context of immune regulation. In this review, we summarize pre-clinical data derived from experimental murine models that have examined the role of both natural and induced Treg cells in the biology of GVHD. We also review the clinical studies which have begun to employ Treg cells as a form of adoptive cellular therapy for the prevention of GVHD in human transplant recipients.

## Graft Versus Host Disease

Although hematopoietic stem cell transplantation (HSCT) has been a successful therapeutic strategy for treating hematological malignancies for several decades, its broad application is limited by the high incidence of graft versus host disease (GVHD). GVHD is primarily a donor T cell-mediated syndrome whereby T cells in the graft elicit an immune response, resulting in host tissue damage (Korngold and Sprent, 1978). HSCT recipients typically receive conditioning regimens consisting of chemotherapy and/or radiation in order to eliminate their underlying malignancy and facilitate the engraftment of allogeneic stem cells. However, the conditioning regimen can cause damage to host tissues, triggering the release of proinflammatory cytokines such as TNF-α, interleukin-1β, and interleukin-6, and activating the innate immune system, including host antigen presenting cells (APCs) (Hill et al., 1997; Shlomchik et al., 1999). Early following transplantation, donor T cells in the graft interact with activated host APCs, recognize presented host peptides as foreign, and differentiate into cytokine-producing T effector cells. The ensuing proinflammatory cytokine storm recruits other effector cells, like NK cells and macrophages. This perpetuates the proinflammatory cytokine cascade that is a hallmark of acute GVHD (aGVHD) and results in direct tissue damage, generally to a restricted set of organs (i.e., skin, liver, and gastrointestinal tract) (Antin and Ferrara, 1992). A second phase of GVHD, known as chronic GVHD (cGVHD) tends to have a more delayed presentation in patients, broader organ involvement, and clinical features that bear strong resemblance to autoimmune disorders (Graze and Gale, 1979). Both aGVHD and cGVHD can be characterized as resulting from an imbalance between the effector and regulatory arms of the immune system (Chen et al., 2009). Clinical approaches that restore effective immune regulation are therefore an attractive treatment strategy for GVHD, which currently has no FDA-approved therapies. To that end, regulatory T (Treg) cells which are potent suppressors of immune responses have been a focal point of research studies designed to mitigate the severity of GVHD in both pre-clinical murine models and in early stage clinical trials. The optimization of these approaches, however, requires a thorough understanding of the various Treg cell subsets and how they coordinately regulate alloreactive donor T cell responses during GVHD.

## CD4^+^ Treg Cell Subsets

In 1995, Sakaguchi et al. (1995) identified a suppressive population of CD4^+^ T cells that expressed high levels of the IL-2 receptor α-chain (CD25). These cells, termed Treg cells, express the forkhead box transcription factor Foxp3, which is both necessary and sufficient for the suppressive ability of Treg cells (Fontenot et al., 2005). Importantly, there are two distinct subsets of CD4^+^ Treg cells. Natural Treg (nTreg) cells comprise 5–10% of the CD4^+^ T cell compartment and develop in the thymus (Sakaguchi et al., 2006). During negative selection, nTreg cells upregulate Foxp3 when they recognize self-antigen rather than undergoing clonal deletion. nTreg cells are responsible for maintaining immune homeostasis and tolerance to self-antigen by inhibiting self-reactive T cells in the periphery (Sakaguchi et al., 2006; Curotto de Lafaille and Lafaille, 2009). A second subset of Treg cells which has been termed induced Treg (iTregs) cells is generated when conventional T cells are activated in the context of TGF-β and IL-2, resulting in the upregulation of Foxp3 (Fantini et al., 2004). Alternatively, iTregs can also be induced in a TGF-β-independent fashion (Schallenberg et al., 2010). Although the role of iTreg cells in controlling the immune response is not completely understood, these cells are thought to be important for regulating peripheral T cell activation during infection and mediating the contraction phase of the immune response (Curotto de Lafaille and Lafaille, 2009). iTreg cells can also be generated *in vitro* by activating naive T cells with either antigen or anti-CD3/anti-CD28 antibodies in the presence of TGF-β and IL-2 (Chen et al., 2003; Fantini et al., 2004). *In vitro*-generated iTreg cells are clinically attractive since they can be grown in large numbers which facilitates the adoptive transfer of these cells into recipients under conditions where obtaining a similar number of nTreg cells may be logistically difficult. The relative roles of nTreg and iTreg cells in regulating immune responses and the extent to which they have unique or overlapping capabilities, however, has not been defined and is an area of active investigation. Studies performed by Haribhai et al. in murine models of colitis or Foxp3-deficiency both suggest that nTreg cells and *in vivo*-derived iTreg cells have distinct roles in preventing disease and that these populations act in a complementary fashion to reduce inflammation (Haribhai et al., 2009, 2011). Elucidating whether a similar relationship exists between these two Treg cell populations in GVHD has not been critically examined.

## Role of CD4^+^ nTreg Cells in Pre-Clinical Models of GVHD

Since GVHD is characterized by the loss of tolerance and the development of autoimmune manifestations, it is reasonable to postulate that a deficiency in Treg cell reconstitution plays a critical role in GVHD pathophysiology. In fact, studies in mice have demonstrated that there is a progressive loss of Treg cells during aGVHD, and this leads to the emergence of autoreactive proinflammatory donor T cells (Chen et al., 2007). These cells are able to mediate pathological damage when re-exposed to self antigens which leads to autoimmunity, a hallmark of cGVHD. Thus, the absence of Treg cells appears to contribute to both aGVHD and cGVHD.

Given the critical role of Treg cells in the maintenance of tolerance, several groups have tested the hypothesis that the adoptive transfer of Treg cells should ameliorate disease by restoring defective tolerance mechanisms. These studies have been typically performed by the isolation of CD4^+^ CD25^+^ T cells from the spleen and secondary lymphoid tissue, or more recently, by obtaining Treg cells from reporter mice in which GFP and Foxp3 proteins are co-expressed in transgenic animals. It should be noted that this population of cells which is generally considered to consist of nTreg cells may actually include some iTreg cells, as there are currently no reliable markers to distinguish the two subsets (Curotto de Lafaille and Lafaille, 2009). However, since iTreg cells must be activated in order to upregulate Foxp3 (Fantini et al., 2004), naïve mice are presumed to have much lower numbers of iTreg cells. Therefore, CD4^+^ Foxp3^+^ T cells isolated from naïve mice have been operationally considered to be nTreg cells.

The first published study was from Taylor et al. (2002) who reported that both depletion of CD25^+^ T cells from the transplant inoculum as well as *in vivo* CD25^+^ T cell depletion after transplantation was associated with worsening of GVHD. In contrast, the adoptive transfer of CD4^+^ CD25^+^ nTreg cells along with the marrow graft resulted in the amelioration of disease. Since nTreg cells are difficult to isolate in large numbers from the spleen and secondary lymphoid tissues, this group *ex vivo* activated and expanded CD4^+^ CD25^+^ T cells, and demonstrated that these expanded nTreg cells were also potent suppressors of GVHD (Taylor et al., 2002). These results were rapidly confirmed by other investigators (Hoffmann et al., 2002; Edinger et al., 2003). Subsequent studies demonstrated that adoptively transferred nTreg cells must be of donor origin and that their suppressive ability was due, at least in part, to IL-10 secretion (Hoffmann et al., 2002; Tawara et al., 2012). Notably, nTreg cell adoptive transfer was most effective when these cells were transferred before or at the time of transplantation, while cell transfer at later time points post transplantation was less effective at attenuating disease severity (Hoffmann et al., 2002; Taylor et al., 2002; Edinger et al., 2003). The critical role for timing derived from the fact that nTreg cells are necessary for inhibiting the early expansion of alloreactive donor T cells (Edinger et al., 2003).

**Figure 1:**
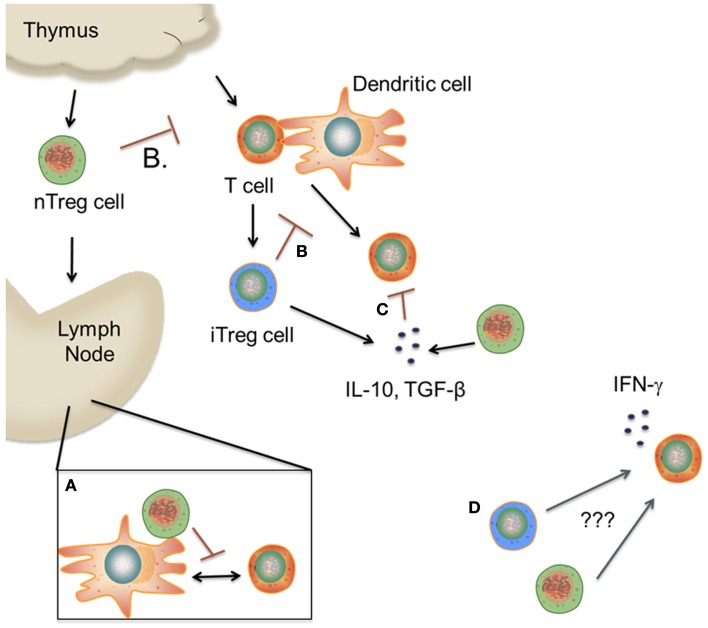
**Proposed mechanism(s) of Treg cell suppression during GVHD**. **(A)**. nTreg cells migrate to secondary lymphoid tissues, where they prevent allorecognition by blocking the interaction between T cells and dendritic cells. **(B,C)** nTreg and iTreg cells inhibit T cell activation in the periphery by various mechanisms including cytokine deprivation, inhibitory receptors, and release of suppressive cytokines. **(D)** A subset of nTreg and iTreg cells lose Foxp3 expression and begin to secrete proinflammatory cytokines due to unknown environmental cues. The role of these cells in mediating pathological damage during GVHD is unknown. (This figure was created using Visi ScienceSlides^®^ Software).

Early post transplantation, nTreg cells migrate to secondary lymphoid organs, where they interact with effector T cells (Nguyen et al., 2007) (Figure 1). Two studies concluded that only CD62L^*hi*^ nTreg cells and not CD62L^*lo*^ nTreg cells were able to mitigate GVHD, suggesting that migration to the spleen and lymph nodes early post transplantation is critical for nTreg cell suppressive function (Taylor et al., 2004; Ermann et al., 2005). This was further evidenced by the fact that CD62L^*lo*^ nTregs were able to suppress alloreactive T cell proliferation *in vitro* but were non-functional *in vivo* (Ermann et al., 2005). Subsequent studies demonstrated that nTreg cells were necessary during T cell priming in order to suppress GVHD-induced CD8^+^ T cell proliferation (Wang et al., 2009) and render CD8^+^ T cells anergic (Kim et al., 2006). A requirement for host antigen presentation on host APCs was also identified to be both necessary and sufficient for nTreg cells to attenuate lethal GVHD (Tawara et al., 2010).

Studies involving chemokine receptor expression on nTreg cells further elucidated the importance of trafficking in nTreg cell-mediated suppression of GVHD. CXCR3, CCR5, and CCR6 are chemokine receptors that are responsible for directing cells toward GVHD target organs (liver, lung, intestine) which are the sites of GVHD-associated tissue damage (Wysocki et al., 2005; Varona et al., 2006; Hasegawa et al., 2008). nTreg cells transfected with CXCR3 display increased protection against GVHD as compared to untransfected nTreg cells (Hasegawa et al., 2008). Similarly, nTreg cells that are either CCR5 or CCR6 deficient exhibit diminished suppressive function *in vivo* despite their potent suppressive function *in vitro*, as they are unable to migrate to sites of inflammation (Wysocki et al., 2005; Varona et al., 2006). Zhao et al. (2008) also reported that CD4^+^ CD103^+^ Foxp3^+^ nTreg cells migrate directly to GVHD target organs due to high expression of CCR5 and low expression of CD62L, and are able to ameliorate cGVHD severity, providing additional confirmation that Treg cell trafficking is critical for optimal protection from GVHD.

Although *ex vivo* nTreg cell adoptive transfer studies have been relatively successful in preventing lethal GVHD, *in vivo* expansion of nTreg cells may provide a more clinically relevant approach for nTreg cell therapy. As previously noted, nTreg cells represent a minor population in the periphery; thus isolating these cells in sufficient numbers for clinical use may be challenging. Furthermore, while *ex vivo* expansion of nTreg cells preserves their suppressive function, conducting clinical protocols that require extended cell culture can be expensive, technically challenging, and difficult to implement in many centers. *In vivo* expansion of nTreg cells is therefore an attractive option when confronted with limited resources for clinical translation. To that end, several pre-clinical studies have demonstrated feasibility of this approach. One strategy has employed IL-6 receptor blockade to increase both nTreg and iTreg cell numbers in animals undergoing GVHD. Mice treated with an anti-IL-6R monoclonal antibody exhibited increased Treg cell reconstitution, decreased proinflammatory cytokine secretion, and improved overall survival (Chen et al., 2009). These studies are particularly relevant given that there is currently an FDA-approved anti-IL-6R antibody, Tocilizumab, which has shown activity in steroid refractory GVHD (Drobyski et al., 2011), although whether this is attributable to an increase in Treg cell numbers awaits further study. An alternative approach has employed a monoclonal anti-CD28 antibody that acts as a superagonist and results in the preferential expansion of nTreg cells and a corresponding mitigation in GVHD severity (Kitazawa et al., 2009).

Pharmacological strategies have also been tested in murine GVHD models to determine whether Treg cell numbers can be augmented after allogeneic HSCT. To that end, Shin et al. (2011) demonstrated that the *in vivo* administration of rapamycin plus IL-2 antibody complexes expanded the nTreg cell population and reduced GVHD severity. Furthermore, a synthetic derivative of a-galactosylceramide (KRN7000) which is a ligand for the CD1d molecule has been shown to expand donor-derived Treg cells in a dose-dependent manner and reduce GVHD-associated mortality (Duramad et al., 2011). It is pertinent to note that it is difficult to distinguish between *in vivo* expansion of nTreg cells and *in vivo* conversion and/or expansion of iTreg cells. Thus, it is difficult to exclude that these approaches may also result in the expansion of iTreg cell populations as well.

## Role of CD4^+^ iTreg Cells in Pre-Clinical Models of GVHD

While the majority of rodent models of GVHD have focused on the biology of nTreg cells, there has been much less attention devoted to the role of iTreg cells in GVHD biology. This has been due, in part, to the fact that there are no proven cell surface markers that distinguish nTreg cells from iTreg cells. Consequently, isolation of a pure iTreg cell population from donor animals for selective adoptive transfer studies is not currently feasible. Furthermore, *de novo* iTreg cell generation in recipient mice is negligible during GVHD (Chen et al., 2009; Beres et al., 2011), making identification and isolation of these cells in the host problematic as well. However, iTreg cells can be easily generated from the conventional T cell pool and expanded in culture (Fantini et al., 2004; Beres et al., 2011). For this reason, the study of iTreg cells during GVHD has been almost exclusively limited to the *in vitro* induction/expansion of this population followed by adoptive transfer into recipient animals. In initial studies, iTreg cells were stimulated with allogeneic dendritic cells or treatment with anti-CD3/anti-CD28 antibodies in the presence of TGF-β and IL-2 to induce Foxp3 expression. Administration of *in vitro*-differentiated iTreg cells along with BM grafts containing alloreactive donor T cells did not result in any significant protection from lethal aGVHD (Koenecke et al., 2009; Beres et al., 2011), although one study did demonstrate efficacy in a lupus-like cGVHD model (Zheng et al., 2004). A major reason for the lack of observed protection in the aGVHD models was the fact that there was limited *in vivo* survival of these cells which was accompanied by instability of Foxp3 expression, resulting in a loss of suppressive function early post transplantation (Koenecke et al., 2009; Beres et al., 2011).

The reason that iTreg cells are unstable *in vivo* is not clear, but one potential explanation is that the proinflammatory cytokine milieu that occurs during GVHD may also render iTreg cells more unstable. Supporting this premise are data demonstrating that *in vivo*-derived iTreg cell conversion is significantly enhanced when mice are treated with monoclonal antibodies that block signaling through IL-6 or IL-21 which serves to reduce inflammatory cytokine production (Bucher et al., 2009; Chen et al., 2009). Notably, both of these cytokines signal through Stat3 and Stat3-dependent cytokines have been reported to limit iTreg cell generation during GVHD (Pallandre et al., 2007; Laurence et al., 2012). Indirect support for this premise also comes from the fact that the only study in which iTreg cells that were generated by allogeneic dendritic cell stimulation were able to mitigate GVHD and maintain their suppressive phenotype (Sela et al., 2011) was one which employed a non-irradiation GVHD model where inflammatory cytokine production is more attenuated. Apart from blocking Stat 3-dependent cytokines as a strategy to augment iTreg cell reconstitution *in vivo*, an alternative approach has involved the culture of CD4^+^CD25^−^ T cells with the hypomethylating agent 5-azacytidine. Choi et al. (2010) reported that this treatment induced Foxp3 expression in conventional CD4^+^ T cells both *in vitro* and *in vivo*, and that transplantation of these cells ameliorated GVHD severity.

We would note that instability of Foxp3 expression has also been noted to occur in nTreg cells in non-transplant models (Zhou et al., 2009; Pillai et al., 2011) as well as in GVHD (Laurence et al., 2012), where these cells can revert to a proinflammatory phenotype under inflammatory conditions. Thus, inflammation appears to affect Foxp3 stability in both CD4^+^ Treg cell populations.

## CD8^+^ Treg Cells in GVHD

Foxp3^+^ Treg cells are classically defined as being a subset of the CD4^+^ T cell compartment. However, a CD8^+^ Foxp3^+^ Treg population has been described and found to be capable of suppressing T cell responses in animal models of autoimmunity and allergen exposure (Hahn et al., 2005; Tsai et al., 2010; Wong et al., 2010). Furthermore, these cells have been shown to play a suppressive role in patients that have undergone autologous HSCT for systemic lupus erythematosus, and robust reconstitution of this cell population has been associated with more durable remissions (Zhang et al., 2009). CD8^+^ Foxp3^+^ T cells have also been documented in the tumor microenvironment of patients with colon and prostate cancer, suggesting that they may be a mechanism by which tumors escape immune surveillance (Kiniwa et al., 2007; Chaput et al., 2009). With respect to GVHD, work by three independent groups reported that a suppressive population of CD8^+^ Foxp3^+^ iTreg cells are induced early during GVHD (Beres et al., 2012; Robb et al., 2012; Sawamukai et al., 2012). Like their CD4^+^ counterparts, these cells were found to be dependent on TGF-β and IL-2 for induction (Sawamukai et al., 2012) and comprised up to 70% of the total iTreg population post transplantation (Beres et al., 2012). Using different methodologic approaches, all three studies also demonstrated that at least one functionally competent CD4^+^ or CD8^+^ iTreg cell population was required to prevent increased GVHD-associated mortality (Beres et al., 2012; Robb et al., 2012; Sawamukai et al., 2012). Interestingly, a small adoptively transferred population of CD8^+^ iTreg cells could be expanded in GVHD recipients using IL-2 antibody complexes in conjunction with Rapamycin as has been previously described with CD4^+^ Treg cells (Shin et al., 2011; Robb et al., 2012). Recently, alloantigen-specific human CD8^+^ Foxp3^+^ T cells have been induced *in vitro* and found to suppress GVHD in a humanized mouse model (Zheng et al., 2012). Protection was associated with a reduction in chemokine and inflammatory cytokine production. These data suggest that these cells may also be relevant in human allogeneic HSCT for the protection from lethal GVHD. Since these cells do not exist in the naïve state, however, they will likely need to be expanded using *in vitro* or *in vivo* methodological approaches for translational application.

## Role of Treg Cells in Human GVHD

The approach that has been employed to address whether Treg cells may serve to modulate the severity of GVHD in man has been to correlate the absolute number and/or frequency of Tregs with the subsequent incidence and severity of aGVHD and cGVHD. Several reports have demonstrated a decreased frequency of Treg cells in the peripheral blood of patients with high clinical grades of aGVHD as compared to patients with lower grade aGVHD or no GVHD (Li et al., 2010; Bremm et al., 2011). Moreover, Treg cell frequency was shown to be reduced by as much as 40% in the peripheral blood of allogeneic HSCT recipients that developed GVHD as compared to autologous or allogeneic HSCT recipients that displayed no signs of GVHD (Magenau et al., 2010). Similar results have also been observed in cGVHD, where the frequency of Treg cells negatively correlated with disease severity (Zorn et al., 2005; Li et al., 2010; McIver et al., 2012). Whereas most human studies have examined peripheral blood Treg cells; Rieger et al. assessed mucosal Treg cell frequencies in intestinal biopsies, which are perhaps a more relevant marker of disease. This group reported that the ratio of Foxp3^+^ Treg cells to CD8^+^ T cells was significantly decreased at the mucosal interface of GVHD patients as compared to patients with intestinal inflammation unrelated to GVHD (Rieger et al., 2006). Although most of these human studies have examined Treg cell frequency several weeks post transplantation after the establishment of GVHD, one report found that the ratio of Treg cells to T cells was decreased in aGVHD patients within 2 weeks of transplantation, prior to disease onset, suggesting that this ratio may also be a good clinical predictor of GVHD (Fujioka et al., 2013).

It is important to note, however, that not all studies have demonstrated a correlation between reduced Treg frequency and GVHD severity. Clark et al. (2004) observed that cGVHD patients had increased numbers of peripheral blood CD4^+^CD25^*hi*^ Treg cells as compared to individuals without GVHD. This was supported by a more recent study that reported increased peripheral Treg cell numbers in transplant recipients that developed cGVHD with no prior aGVHD diagnosis (Ukena et al., 2011a). Interestingly, the same study found decreased peripheral blood Treg cell frequencies in patients whose aGVHD transitioned into cGVHD, although the frequency of Treg cells in these patients increased over a 6-month observation period (Ukena et al., 2011a). Treg cells isolated from the peripheral blood of GVHD patients were also found to display normal suppressive function (Clark et al., 2004; Noel et al., 2008). Arimoto et al. (2007) employed an alternative strategy and demonstrated no significant correlation between Foxp3 expression and the incidence of either aGVHD or cGVHD, as measured by mRNA isolated from peripheral blood lymphocytes from allogeneic HSCT recipients. Finally, gastric biopsies had comparable mucosal Treg cell frequencies in patients with gastric GVHD and patients with no GVHD, suggesting that Treg cell frequencies do not correspond to disease incidence or severity in this tissue site (Lord et al., 2011).

The reason for the differences observed in these studies is not entirely clear. For the most part, however, studies that have failed to demonstrate that a reduction in Treg cell frequency and/or absolute numbers is associated with increased GVHD severity have relied on CD25 expression to delineate Treg cell populations, whereas those that have reported a positive correlation have tended to employ Foxp3 expression as a readout for this Treg cell population. Thus, it is possible that the reliance on different phenotypic markers may result in somewhat different populations being examined and be a potential explanation for these discordant results.

## Donor-Derived Treg Cells in Human HSCT

An alternative approach to examine the effect of Treg cells on GVHD severity in human allogeneic HSCT has been to assess the number of donor-derived Treg cells within the graft prior to transplantation. In this regard, Rezvani et al. (2006) determined that increased frequencies of CD4^+^Foxp3^+^ Treg cells in the peripheral blood of the donor negatively correlated with the incidence of GVHD in the graft recipient. Several subsequent studies confirmed this correlation in recipients of HLA-identical sibling and unrelated donor stem cell grafts (Pabst et al., 2007; Wolf et al., 2007), indicating that hematopoietic stem cell graft content appears to modulate GVHD severity. Notably, Blache and colleagues reported that although peripheral blood stem cell (PBSC) grafts include increased numbers of CD4^+^CD25^+^CD127^*lo*^ Treg cells as compared to bone marrow grafts, the frequency of peripheral blood Treg cells is reversed post transplantation. This was presumed to be due to the fact that PBSC Treg cells tended to be CD62L^*lo*^ as a consequence of both granulocyte colony stimulating factor treatment for mobilization and the subsequent leukapheresis process (Blache et al., 2010). In that regard, increased numbers of CD62L^+^ Treg cells in the graft have been found to correlate with reduced GVHD incidence (Lu et al., 2011), which is likely due to the ability of these cells to enter the secondary lymphoid tissue where allorecognition by donor T cells and GVHD initiation occurs. This is consistent with what has been reported in rodent models of GVHD where the CD62L^+^ Treg cell population is more potent at suppressing GVHD than the corresponding CD62L^*lo*^ population (Taylor et al., 2004).

## Treg Cell Clinical Trials

Less than two decades after their discovery, Treg cells are now entering into clinical trials in allogeneic HSCT recipients. Pre-clinical murine models of GVHD have provided much insight into Treg cell-based therapy, but most mouse studies have been performed using Foxp3-GFP reporter mice where Foxp3-expressing Treg cells can be definitively isolated for adoptive transfer studies. This is not a luxury that is available in human studies where CD25 expression necessarily serves as a surrogate for Foxp3. However, since CD25 is upregulated on all activated T cells, further phenotypic characterization of these cells has been generally thought to be necessary for their use in man. To that end, Ukena et al. (2011b) compared the phenotype, function, and stability of many Treg cell subsets and deemed that CD4^+^CD25^*hi*^ CD127^−^ or CD4^+^CD25^*hi*^ICOS^+^ Treg populations were likely to be most suitable for human adoptive transfer studies. Many groups have also identified *in vitro* expansion protocols that yield high number of Treg cells for adoptive transfer (Karakhanova et al., 2006; Hippen et al., 2011a,b; Veerapathran et al., 2011; Chakraborty et al., 2013; Golab et al., 2013). Recently, Hippen et al. (2011a) utilized rapamycin and TGF-β treatment to generate and expand iTreg cells, which were potent suppressors in a xenogeneic model of GVHD. Likewise, Chakraborty et al. defined a protocol for the large-scale expansion of nTreg cells. These *ex vivo*-expanded cells also ameliorated disease in a xenograft model of GVHD (Chakraborty et al., 2013).

One of the first reported clinical studies was conducted by Brunstein et al. who performed a phase I clinical prophylaxis trial with cord blood-derived Treg cells. The rationale for the use of cord blood-derived Treg cells was based, in part, on earlier studies, that had shown that they express similar levels of CTLA-4, Foxp3, GITR, and CD25 as adult peripheral blood Treg cells, and when stimulated by alloantigen, were potent suppressors of T cell expansion (Chang et al., 2005). Furthermore, cord blood Treg cells were shown to be resistant to immunosuppressant drugs that are commonly used to treat GVHD (Porter et al., 2006) and could therefore interfere with Treg suppressive function. In this phase I trial, Brunstein et al. (2011) demonstrated that these cells could be safely administered, but whether they had a role in protecting patients from GVHD could not be adequately assessed due to the design of the study.

A second clinical trial was performed by Di Ianni et al. (2011), in which Treg cells were adoptively transferred into patients receiving haploidentical transplants. This was also a prophylaxis trial but the source of Treg cells was from the donors who also provided the stem cells that were used to engraft the patients, as opposed to cord blood. In this report,>90% of enrolled patients engrafted and only 2/26 patients had ≥grade 2 aGVHD. No patient had developed cGVHD at the time of publication.

There has been one small study involving two patients in which expanded Treg cells were administered to patients with documented GVHD (Trzonkowski et al., 2009), as opposed to being given to prevent disease. Cells were obtained from family donors who were HLA-identical with the recipients, activated with anti-CD3/CD28 beads and then cultured in high doses of IL-2 for 3 weeks. One patient had cGVHD, while the second had refractory aGVHD. The former patient had a partial response as determined by the ability to reduce concurrent immune suppressive agents along with objective improvement in some clinical parameters. The latter patient, however, had no sustained improvement despite multiple Treg cell infusions.

An alternative approach to harness the potential for Treg cell therapy in humans is based on the requirement of these cells for IL-2. Specifically, Zorn et al. (2009) found that IL-2 therapy in combination with CD4^+^ donor leukocyte infusions, used to treat relapsed hematologic malignancies post HSCT, resulted in Treg cell expansion *in vivo*. This same group then utilized this strategy to treat glucocorticoid refractory cGVHD patients. The administration of low dose IL-2 was associated with an amelioration of disease severity and this correlated with an increase in the number of Treg cells (Koreth et al., 2011). Thus, the administration of cytokines capable of inducing the *in vivo* expansion of Treg cells may be a more clinically feasible strategy to enhance Treg reconstitution post transplantation, as compared to more costly expansion strategies.

Collectively, these studies are exciting evidence that Treg cell therapy has now entered into the clinic. Going forward, well-designed trials will be necessary to determine whether these cells are indeed capable of preventing and/or treating patients with established GVHD.

## Unresolved Questions

Despite the significant progress that has been made in understanding the role of Treg cells in GVHD biology, a number of questions remain. First of all, the relative roles of CD4^+^ nTreg cells, CD4^+^ iTreg cells, and CD8^+^ iTreg cells in GVHD biology remain unclear. Elucidating the mechanisms by which the respective cell subsets function may provide insight for developing better therapeutic strategies. Furthermore, additional studies are required to ascertain whether CD4^+^ and CD8^+^ Treg cell populations function cooperatively or whether they have overlapping redundant roles in GVHD biology.

Secondly, accumulating evidence indicates that Treg populations, particularly those that are expanded *in vitro*, have unstable Foxp3 expression. Since Foxp3 expression is necessary for suppressive function, further inquiry is needed to determine whether Foxp3 expression can be stabilized especially under pro inflammatory conditions which characterizes the GVHD milieu (Koenecke et al., 2009; Beres et al., 2012; Laurence et al., 2012).

Finally, current Treg cell-based immunotherapy approaches rely on the expansion of polyclonal populations of Treg cells. The best source of Treg cells and the optimal culture conditions for *ex vivo* expansion remain unresolved. Moreover, it is possible that alloantigen-specific Treg cells may be more potent in suppressing GVHD, and should be studied further (Albert et al., 2005; Gaidot et al., 2011; Sagoo et al., 2012). A potential advantage of this strategy is that adoptively transferred Treg cells may not suppress the immune response to third party antigens which could preserve the ability of patients to mount competent anti-infectious and anti-tumor immunity (Gaidot et al., 2011).

## Conflict of Interest Statement

The authors declare that the research was conducted in the absence of any commercial or financial relationships that could be construed as a potential conflict of interest.
